# Does the Presence of Anxiety and Depression Symptoms Mediate the Association Between Family Functions and Self-Efficacy in Pregnant Women in the Third Trimester?: A Community-Based Cross-Sectional Survey

**DOI:** 10.3389/fpsyt.2021.726093

**Published:** 2021-11-04

**Authors:** Zhou Wensu, Zhu Xidi, Li Shaojie, Zheng Baohua, Yu Yunhan, Xu Huilan, Hu Zhao, Xiong Xiyue

**Affiliations:** ^1^NHC Key Laboratory of Birth Defect for Research and Prevention, Hunan Provincial Maternal and Child Health Care Hospital, Changsha, China; ^2^Department of Child Rehabilitation, Hunan Provincial Maternal and Child Health Care Hospital, Changsha, China; ^3^Department of Social Medicine and Health Management, Xiangya School of Public Health, Central South University, Changsha, China; ^4^Department of Epidemiology and Health Statistics, Xiangya School of Public Health, Central South University, Changsha, China

**Keywords:** depression symptoms, anxiety symptoms, self-efficacy, family function, pregnancy, mediation modeling

## Abstract

**Objectives:** Pregnant women in the third trimester can be more vulnerable to adverse mental health outcomes, but there is limited research on the association between family function and self-efficacy and potential mediation by symptoms of anxiety and depression.

**Methods:** The cross-sectional study enrolled 813 pregnant women in the third trimester from 14 communities of Hengyang city, Hunan province of China. All of the participants completed a battery of self-report measures of family function (Family Adaptation Partnership Growth and Resolve Index, APGAR-family), self-efficacy (General Self-Efficacy Scale, GSES), anxiety (measured by Generalized Anxiety Disorder scale with seven items, GAD-7), and depression symptoms (Patient Health Questionnaire with nine items, PHQ-9). The correlation analysis was performed using Spearman's correlation coefficient. Baron and Kenny's method and multiple mediation models with bootstrapping were used to determine whether the symptoms of anxiety and depression mediated the association between family functions and self-efficacy.

**Results:** There were 22.6% pregnant women in the low level of self-efficacy, with 60.9% in the moderate level and 16.5% of respondents in the high level. Self-efficacy had significant correlations with both anxiety symptoms (*r* = −0.19, *p* < 0.05), depression symptoms (*r* = −0.22, *p* < 0.05), and family function (*r* = 0.31, *p* < 0.05). Anxiety symptoms were significantly associated with self-efficacy (β = −0.016, *p* < 0.05). Depression symptoms were significantly associated with self-efficacy (β = −0.024, *p* < 0.05). Anxiety and depression symptoms partly mediated the association between family function and self-efficacy, accounting for 11.4 and 16.4% of total effect, respectively. It was indicated that pregnant women with a high degree of family function are less likely to have emotional symptoms and predicted to have higher levels of self-efficacy.

**Conclusions:** Anxiety and depression show mediating effects in the association between family function and self-efficacy. Improved family function can have a positive impact on pregnant women in the third trimester.

## Introduction

Self-efficacy is defined as an individual's ability to change his/her behavior (1). To be specific, it is one aspect of confidence that a person believes in his/her abilities and competencies for achieving success (2). Research has shown self-efficacy to be negatively correlated with anxiety and depression symptoms but positively correlated with family support (3, 4). For pregnant women, higher self-efficacy is related to higher confidence in infant care practices, while inadequate self-efficacy can lead to perceived barriers, breastfeeding disorders, lack of health education program compliance (5), high or severe fear of childbirth, or tokophobia (6). A low level of self-efficacy also poses serious threats to successfully coping with labor and the quality of life of pregnant women (7).

Family functions, which are closely related to social support, global stress, and mental health perception in women, emphasize the family as a multidimensional concept that includes the effectiveness of family member's emotional connection, family regulation, family communication, and coping with external events in life (8, 9). Family functions are more important for pregnant women, especially in families with a Confucian cultural context that is widely located in Asia and other countries (10, 11). Moreover, these families emphasize family partnership and family affection to provide full support to pregnant women and adversities that they may face. However, more expectations and perceptional gaps devised from family members, especially eldership for a new baby, may be a matter of conflict. The importance of a positive role for well-Functioning families to prevent negative psychological outcomes has also been discussed in a previous study (12).

Existing studies have shown that the prevalence of antenatal depression and anxiety symptoms in pregnant women is common worldwide (13). Observational data suggested a U-shaped relationship between psychological problems and the first, second, and third trimesters of pregnancy (14). It is now increasingly acknowledged that pregnant women in the third trimester experience more stress due to factors such as rapid development of the fetus, beginning of labor, worry about child care, and family relationships (15). In a sample from the Netherlands, the prevalence of depression and anxiety symptoms in the last trimester was approximately 10.0 and 14.2%, respectively (16). In China, ~17.4% of pregnant women suffer from depressive symptoms during pregnancy (17). The prevalence of antenatal depression is found to be higher in high-income countries (HICs) than in low- and middle-income countries (LMICs) (18). Antenatal emotional symptoms are harmful to mothers as well as their offspring (19). Several studies have summarized that depression or anxiety symptoms during pregnancy can produce a range of neurodevelopmental outcomes in children from lower birth weight for gestational age to increased probability of extreme psychological problems later in life (e.g., psychotic experiences). Furthermore, these pregnant women are more likely to face early delivery and pregnancy-associated diseases compared to others (20, 21).

To date, evidence suggests that low levels of family function and self-efficacy are significantly related to emotional problems in pregnant women (22). In other words, function may intensify resilience to, and coping with, stressors (11) and eliminate negative emotional symptoms. Several surveys have found self-efficacy mediated family functioning, depression, and anxiety symptoms in individuals (23, 24). However, few studies have explored the relationship and mechanism among these three factors in pregnant women. Furthermore, emotional problems are also considered precursors for self-efficacy decline (25). These reviews suggest a possible connection among family function, emotional problems, and self-efficacy, which needs further clarification. Wernand et al. (26) reported that anxiety symptoms can uniquely predict parenting self-efficacy during pregnancy, consistent with another study on adult women (27). A comprehensive theoretical model (McMaster Model of Family Functioning) suggested that family member's mental health can be well-maintained only when the basic functions of the family are realized (28). Thus, we believed that it would be possible for a family function to influence an individual's self-efficacy, and this association might be further mediated by symptoms of anxiety and depression.

Women are more vulnerable and susceptible to psychological problems due to hormonal and/or biological changes during pregnancy (29). The interaction effect on psychological problems has been established as a crucial factor for pregnant women's health. Therefore, based on a community-based survey from a representative mid-income country, this study was planned to resolve the following three research questions: (1) What is the status of emotional problems, family functioning, and self-efficacy among pregnant women in the third trimester? (2) What is the relationship between emotional problems, family functioning, and self-efficacy? (3) How do anxiety or depression symptoms mediate the relationship between family function and self-efficacy? We also provided public health implications to identify vulnerable populations among pregnant women and identify effective measures for mothers and infants to improve maternal quality during pregnancy and postpartum recovery.

## Methods

### Data Source

**Figure 1:**
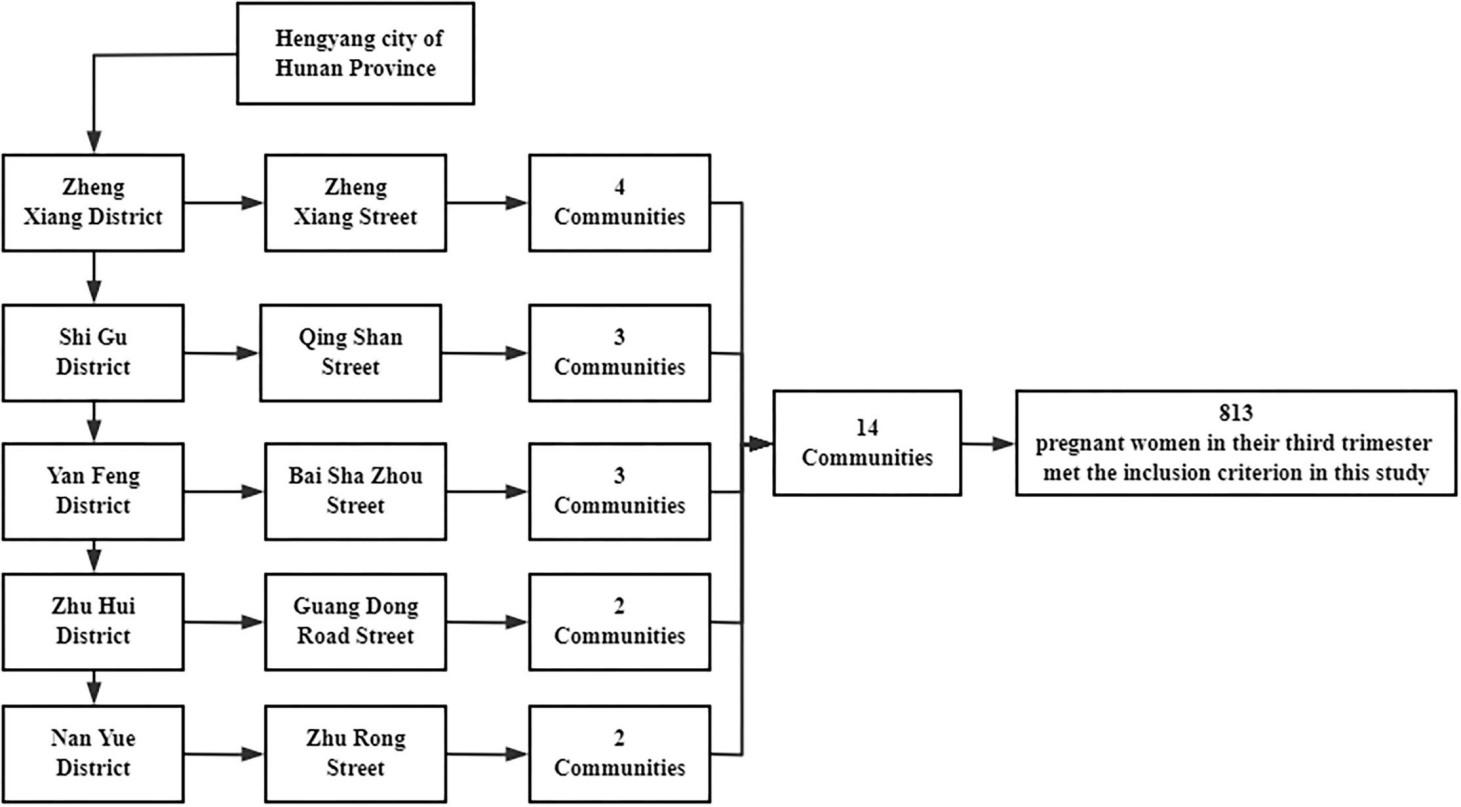
Sampling strategy for the 14 communities of Hengyang city.

This was a community-based cross-sectional survey between July 2019 and September 2019, which was conducted in Hengyang city, Hunan province, China. First, we selected five streets from five administrative districts in the city by stratified sampling. We then randomly selected few communities from each street as follows: four communities from Zhengxiang street, three communities from Qingshan Street, three communities from Baishazhou Street, two communities from Guangdong Road Street, and two communities from Zhurong Street. The inclusion criteria for the study were (1) pregnant women in their third trimester with the pregnancy registered in the community health centers, (2) those participants were not diagnosed with anxiety/depression disorders by grade II and III hospital before (based on DSM-5), (3) those who volunteered to participate in this project, and (4) those who lived in the community for more than 6 months. All scales and measures were combined into a self-designed questionnaire. Each participant was interviewed for approximately 20 min by trained graduate staff from Central South University, Xiangya School of Public Health. A total of 819 pregnant women were enrolled in our study; however, due to incomplete data, six participants were excluded. Finally, 813 pregnant women with complete information on the third trimester completed the survey, and all of them provided written informed consent. The study was approved by the Ethics Committee of the Xiangya School of Public Health (XYGW-2019-056). The flow chart of this study is showed in Figure 1.

### Family Function

The Family Adaptation Partnership Growth and Resolve (APGAR-family) Index (30) was used to assess family functioning in pregnant women. The scale is composed of five items with total scores ranging from 0 to 10. Each opinion was rated on a three-point scale (2 = always; 1 = sometimes; 0 = almost not). As recommended, the cutoff scores for the APGAR-family index are as follows: 7–10 (good family function), 4–6 (moderate family function disorder), and 0–3 (severe family function disorder). Cronbach's α-coefficient was 0.876 in this study.

### Self-Efficacy

Self-efficacy was measured using 10 items from the General Self-Efficacy Scale (GSES). The Chinese version of the GSES was developed by Zhang and referred to as German Health Psychology and Clinical Expert Scale (31). Each item was rated on a four-point scale (1 = totally incorrect, 2 = moderately correct, 3 = mostly correct, and 4 = completely correct). After dividing the total score of the scale by 10, the score was categorized into three groups: low (0–2), medium (2.1–3), and high (3.1–4) levels. The α-coefficient for the 10-item scale was 0.898.

### Emotional Problems

Emotional problems included anxiety and depression symptoms, which were measured using the Generalized Anxiety Disorder Scale with seven items (GAD-7) and the Patient Health Questionnaire with nine items (PHQ-9), respectively. The GAD-7 is a simple tool with seven items to screen anxiety symptoms based on the Diagnostic and Statistical Manual of Mental Disorders, 4th edition (DSM-IV). Each opinion was four-Likert scored on a scale of 0 (none), 1 (a few days), 2 (more than half the time), or 3 (almost every day) with total scores ranging between 0 and 21. The participants were asked if they had experienced any of the items included during the last 2 weeks. The PHQ-9 has nine items that are widely used to assess depressive symptoms, with a total score ranging between 0 and 27. Each item had four answers (not at all, several days, more than half the days, and nearly every day), and the score varied from 0 to 3 points. Higher scores on both GAD-7 and PHQ-9 represented more severe depressive or anxiety symptoms. The Chinese versions of the GAD-7 and PHQ-9 are widely applied to epidemiological investigations and are acceptable with good validity and reliability among the Chinese population (32). As recommended by previous studies (33, 34), we used 10 points as the cutoff to discriminate anxiety or depression symptoms from no symptoms. In our study, Cronbach's α-coefficient was 0.882 for the GAD-7 and 0.773 for the PHQ-9. To be specific, usually, the GAD-7 and PHQ-9 were used to evaluate the emotional symptoms of the participants. They are developed based on DSM-5. Usually, the GAD-7 and PHQ-9 are separately applied for making criteria-based diagnoses of depressive and anxious disorders commonly encountered in primary care. However, they are primarily screening instruments and the results should be discussed with a psychiatrist or therapist.

### Covariates

The following covariates were chosen as potential confounding variables. Information on several demographic characteristics including age (≤ 24, 25–29, 30–34, and ≥35 years), residence (rural and urban), marital status (married and divorced/widowed/single), employment (yes and no), education (college or above and high school or below), and per-capita monthly income (≥8,000, 3,001–7,999, and ≤ 3,000 RMB) were collected in our study. Present lifestyle behaviors including smoking status (never, current, and quit), alcohol consumption at present (yes or no), and exercising habits at present (yes or no) were considered as covariates in this study. In addition, history of abortion (yes and no), number of child in the family (0, 1, and 2), pregnancy complications (yes and no), and regular obstetrical examination during pregnancy (yes and no) were entered into analysis.

### Statistics Analyses

Demographic characteristics are presented as the mean and standard errors (for continuous variables followed normal distribution) or median and interquartile range (for continuous variables not followed normal distribution) or frequency distributions (for categorical variables). Correlations between emotional problems, family function, and self-efficacy were examined. The mediation tests used (35) four-step indirect effect method by examining whether the effects of family function (the independent variable) on self-efficacy (the dependent variable) operated through a third variable, which is anxiety or depression symptoms (the mediator) after controlling for the effects of covariates (age, education, employment, marital status, per-capita monthly income, smoking status, alcohol consumption at present, history of abortion, number of child in the family, pregnancy complications, regular obstetrical examination during pregnancy, and present exercise habit). According to this criterion, firstly, we examined the significant association between family functions and self-efficacy (path c). Second, analysis for significant association between the mediators and family functions was performed (path a). Then, we established that self-efficacy is associated with the mediators and family functions (path b). We also reported the reverse correlation between the mediators, self-efficacy, and family functions when family functions were considered as dependent variables. The mediation analysis was performed by the multiple linear regression analysis using enter methods. The mediated effect (a × b) and the mediated proportion (a × b/c) were reported in the result. The four steps were all performed after controlling for confounding variable effects. Self-efficacy, family function, depression, and anxiety symptoms were all used as continuous variables in the mediation analysis. Subsequently, a bootstrapping test (*n* = 5,000) was used for mediation effect in the association among depression and anxiety symptoms, family function, and self-efficacy. All analyses were computed using the PROCESS module in the SPSS (SPSS Inc., Chicago, IL, USA) version 25.0. All *p*-values were two-sided, and *p* < 0.05 was considered significant. We also assessed beta (β)–unstandardized beta coefficient and standard deviation (SD) for estimates.

## Results

### General Information on Participants

The mean age of the 813 participants was 29.0 years (*SD* = 4.5) (range, 17–54 years). Of these, 73.2% lived in urban areas. Over half (58.1%) of them had college or higher degrees, 78.7% had permanent jobs, and 95.4% were married; 36.0% of the participants reported to have had a history of abortion. Most of them had one child in their family. Only 10.5% of them had pregnancy complications. Nearly all of them received routine regular obstetrical examination during pregnancy. A total of 70.4% had per-capita monthly income between RMB 3,001 and 7,999 RMB, which was higher than the per-capita disposable income in 2019 of that city (2,351 RMB) (36). Profiles of all participants are presented in Table 1.

**Table 1:** Demographic characteristic and anxiety and depression symptoms of pregnant women.

**Variables**	**Group**	***n* (%)**
Age	≤ 24	115 (14.2)
	25–29	366 (45.0)
	30–34	232 (28.5)
	≥35	100 (12.3)
Residence	Rural	218 (26.8)
	Urban	595 (73.2)
Marital status	Married	776 (95.4)
	Single/divorced/widowed	37 (4.6)
Employment	Yes	599(73.7)
	No	214 (26.3)
Education	College or above	472 (58.1)
	High school	183 (22.5)
	Junior school or below	158 (19.4)
Per-capita monthly income	≥8,000 (RMB)	168 (20.7)
	3,001–7,999 (RMB)	572 (70.3)
	≤ 3,000 (RMB)	73 (9.0)
Smoking status	Never	772 (94.9)
	Quit	38 (4.7)
	Current	3 (0.4)
Alcohol consumption at present	No	733 (90.2)
	Yes	80 (9.8)
Exercising at present	No	70 (8.6)
	Yes	743 (91.4)
History of abortion	No	520(64.0)
	Yes	293(36.0)
Number of child in the family	0	338(41.6)
	1	413(50.8)
	2	62(7.6)
Pregnancy complications	No	728(89.5)
	Yes	85(10.5)
Regular obstetrical examination during pregnancy	No	70(8.6)
	Yes	743(91.4)
Depression symptoms	Yes	75(9.2)
	No	738(90.8)
Anxiety symptoms	Yes	64 (7.9)
	No	749 (92.1)
APGAR	Severe family function disorder	66 (8.1)
	Moderate family function disorder	256 (31.5)
	Good family function	491 (60.4)
GSES	Low level	184(22.6)
	Moderate level	495(60.9)
	High level	134(16.5)

### Self-Efficacy, Family Function, and Emotional Problems Among the Participants

Table 1 shows the measurement scores of 813 respondents. The median and IQR for self-efficacy, family function, depression, and anxiety symptoms were 2.50 (P_25_: 2.10, P_75_: 3.00), 8.00 (P_25_: 5.00, P_75_: 10.00), 4.00 (P_25_: 2.00, P_75_: 7.00), and 2.00 (P_25_: 0.00, P_75_: 6.00), respectively. There were 60.4, 31.5, and 8.1% of participants with good family function, moderate family function disorder, and severe family function disorder, respectively. Of the 813 participants, 22.6% had low levels of self-efficacy, 60.9% had moderate levels, and 16.5% had high levels. The positive rates of depression and anxiety symptoms were 9.2% [95% confidence interval (CI): 7.2–11.2%] and 7.9% (95% CI: 6.6–9.7%) of the participants, respectively. Spearman's correlations showed emotional symptoms significantly associated with self-efficacy (*r* = −0.22, *p* < 0.05 for depression symptoms, *r* = −0.19, *p* < 0.05 for anxiety symptoms) and family function (*r* = −0.28, *p* < 0.05 for depression symptoms, *r* = −0.32, *p* < 0.05 for anxiety symptoms), respectively. Self-efficacy was significantly correlated with family function (*r* = 0.31, *p* < 0.05) (Table 2).

**Table 2:** Spearman's correlations between study variables.

**Spearman's correlations**	**Depression symptoms**	**Anxiety symptoms**	**GSES**	**APGAR**
Depression symptoms	1.00			
Anxiety symptoms	0.63^**^	1.00		
GSES	−0.22^**^	−0.19^**^	1.00	
APGAR	−0.28^**^	−0.32^**^	0.31^**^	1.00

*p < 0.1;

** *p < 0.05;*

***p < 0.01.

### Effect of Family Function and Emotional Problems on Self-Efficacy and the Mediation Role of Emotional Problems

We used (35) four-step test to examine the mediating role of emotional problems in the association between self-efficacy and family function. The outcomes of the hierarchical regression analysis are listed in Tables 3, 4. After controlling for respondent's age, marital status, education, residence, employment, per-capita monthly income, smoking status, alcohol consumption at present, exercise habits, history of abortion, number of child in the family, pregnancy complications, and regular obstetrical examination during pregnancy, we found that family function was significantly related to self-efficacy (β = 0.053, *SE* = 0.008, *p* < 0.05) and depressive symptoms (β = −0.360, *SE* = 0.049, *p* < 0.05), respectively. Family function (β = −0.024, *SE* = 0.006, *p* < 0.05) and depression symptoms (β = 0.045, *SE* = 0.008, *p* < 0.05) were significantly associated with self-efficacy. Similarly, we also observed that anxiety symptoms were significantly associated with family function (β = −0.378, *SE* = 0.054, *p* < 0.05). Family function (β = 0.047, *SE* = 0.008, *p* < 0.05) and anxiety symptoms (β = −0.016, *SE* = 0.005, *p* < 0.05) were significantly associated with self-efficacy scores. Detailed coefficients and standard errors of all variables are presented in Appendices 1, 2.

**Table 3:** Mediation model showing role of depression symptom on relationship between family function and self-efficacy, β (SE), and 95%CI for β.

	**Dependent variable^***a***^**			
	**Path c**	**Path a**	**Path b**	**Inverse path**
**Variables**	**GSES**	**Depression**	**GSES**	**APGAR**
GSES				0.890^***^ (0.156)
				95%CI: 0.584~1.195
Depression			−0.024^***^ (0.006)	−0.152^***^ (0.024)
			95% CI:−0.035~−0.013	95% CI:−0.200~−0.104
APGAR	0.053^***^(0.008)	−0.360^***^(0.049)	0.045^***^(0.008)	
	95% CI:0.038~0.068	95% CI:-0.455~−0.265	95% CI:0.029~0.060	
Constant	2.390^***^ (0.165)	10.158^***^ (1.046)	2.636^***^ (0.172)	5.528^***^ (0.853)
Observations	813	813	813	813
*R* ^2^	0.139	0.108	0.159	0.220
Adjusted *R*^2^	0.117	0.085	0.137	0.200
Residual Std. Error	0.520 (df = 792)	3.307 (df = 792)	0.514 (df = 791)	2.296 (df = 791)
*F* Statistic	6.375^***^ (df = 20; 792)	4.789^***^ (df = 20; 792)	7.127^***^ (df = 21; 797)	10.650^***^ (df = 21; 791)

a *p < 0.1*;

^**^ *p < 0.05;*

*** *p < 0.01.*

*Adjusted for age, marital status, education, residence, employment, per-capita monthly income, smoking status, alcohol consumption, history of abortion, number of child in the family, pregnancy complications, regular obstetrical examination during pregnancy, and exercising at present. Coefficients of confounding variables are presented in Appendix 1*.

**Table 4:** Mediation model showing role of anxiety symptom on relationship between family function and self-efficacy, β (SE), and 95% CI for β.

	**Dependent variable^***a***^**			
	**Path c**	**Path a**	**Path b**	**Inverse path**
**Variables**	**GSES**	**Anxiety**	**GSES**	**APGAR**
GSES				0.936^***^ (0.155)
				95% CI: 0.632 to 1.240
Anxiety			−0.016^***^ (0.005)	−0.131^***^ (0.022)
			95% CI: −0.026 to −0.006	95% CI: −0.174 to −0.088
APGAR	0.053^***^ (0.008)	−0.378^***^ (0.054)	0.047^***^ (0.008)	
	95% CI: 0.038 to 0.068	95% CI: −0.484 to −0.271	95% CI: 0.032 to 0.063	
Constant	2.390^***^ (0.165)	7.792^***^ (1.170)	2.514^***^ (0.168)	4.914^***^ (0.829)
Observations	813	813	813	813
*R* ^2^	0.139	0.111	0.150	0.218
Adjusted *R*^2^	0.117	0.089	0.127	0.197
Residual Std. Error	0.520 (df = 792)	3.699 (df = 792)	0.517 (df = 791)	2.300 (df = 791)
*F* Statistic	6.375^***^ (df = 20; 792)	4.953^***^ (df = 20; 792)	6.634^***^ (df = 21; 791)	10.502^***^ (df = 21; 791)

a *p < 0.1*;

^**^ *p < 0.05;*

*** *p < 0.01.*

*Adjusted for age, marital status, education, residence, employment, per-capita monthly income, smoking status, alcohol consumption, history of abortion, number of child in the family, pregnancy complications, regular obstetrical examination during pregnancy, and exercising at present. Coefficients of confounding variables are presented in Appendix 2*.

The depression and anxiety symptoms served as mediators in the association between self-efficacy and family function, which accounted for 16.4 and 11.4% of the total effect, respectively. A bootstrapping test with 5,000 repetitions was performed to enable the reliability of the analysis. These results suggested that the mediation effect of depression and anxiety symptoms on the association between family function and self-efficacy remained significant (Table 5).

**Table 5:** Bootstrap test (*n* = 5,000) for mediation effect of in the association between emotional problems family function and self-efficacy^*^.

	**Bootstrap test for mediation (Anxiety)**			**Bootstrap test for mediation (Depression)**		
	**Estimate**	**SE**	**95% CI**	**Estimate**	**SE**	**95% CI**
ACME	0.006	0.002	**0.002**–**0.010**	0.008	0.002	**0.004**–**0.013**
ADE	0.050	0.008	**0.035**–**0.065**	0.048	0.008	**0.033**–**0.063**
Total Effect	0.055	0.008	**0.041**–**0.070**	0.055	0.008	**0.041**–**0.070**
Partially standardized indirect effects	0.010	0.004	**0.003**–**0.018**	0.014	0.004	**0.007**–**0.022**

Bold is statistically significant. *Adjusted for age, marital status, education, residence, employment, per-capita monthly income, smoking status, alcohol consumption, history of abortion, number of child in the family, pregnancy complications, regular obstetrical examination during pregnancy, and exercising at present.

## Discussion

This study was a community-based survey to assess self-efficacy, depression and anxiety symptoms, and family function in pregnant women in the third trimester and demonstrated the role of depression and anxiety symptoms in mediating the association between self-efficacy and family function. The results provided evidence for implementing family function interventions to reduce emotional symptoms and enhance self-efficacy. It was a meaningful topic that allowed the results to be generalized to similar regions with the same cultural background to identify high-risk pregnant women with mental health problems.

In line with a study conducted in Taiwan (37), our study also reported that most of the participants had moderate to high levels of self-efficacy and good family function. The status of family function was better than that of other investigations focused on low-income and unmarried couples (38). The prevalence of depression symptoms was lower than that in a study conducted in the US where 27% of pregnant women in the third trimester suffered from moderate-to-severe depression symptoms (39). Furthermore, it was lower than a study where the proportion of pregnant women with depression symptoms was 14.8% (40). A similar difference was observed in a study conducted in other regions of China (41). The positive rate of anxiety symptoms was also lower than (11.1%) that reported by (42). In China, living with family members (especially the parents) is common; thus, women are more likely to spend more time to face family members directly by Ma et al. (43). They may have more family problems to deal with compared with Western women. In our study, we observed that over half of the participants had an education level of college or above; the majority of them were married and had a good income level; more importantly, they were recruited from a city area. Thus, the population in our study had a relatively high socioeconomic status (SES). They may have good ability to eliminate emotional symptoms and have enough resources to deal with challenges. The results underlined that individuals with higher SES would receive more beneficial effects of social capital on health, while socioeconomically deprived people generally would not have enough social capital to produce an effective advantage for health (44, 45).

Correlation analysis showed that family function, depression and anxiety symptoms, and self-efficacy had significant relationships with each other (5, 11, 19). In line with previous reports, our study has summarized that family function protected individuals from negative emotional symptoms and reduced the possibility of forming negative emotional symptoms. A correlation was observed in a sample of children, adolescents, and general women (46–49). Troutman and Recca have shown a negative correlation between depressive symptoms and self-efficacy (50, 51). Our study demonstrated that depression and anxiety symptoms mediated the association between family function and self-efficacy. The mediation effect of depression and anxiety symptoms accounted for 16.1 and 10.9% of the total effect, respectively, and both results indicated partial mediation. This implied that pregnant women with high levels of family function probably could experience fewer emotional symptoms, which could enhance their self-efficacy. Family function plays an irreplaceable role in the field of an individual's life, especially in a family with a solid Confucian cultural background, such as in China, Japan, and Singapore. Families with traditional Saudi culture also emphasize the importance of the family (52). A family not only provides economic support, education, functions of production, substance support, and pension but also brings psychological comfort and support to family members, including the health of all family members (11). Additionally, it promotes the development of the whole society. Pregnant women in the third trimester would more likely to experience a series of physical and psychological symptoms, such as pain, disordered sleep, and depressive symptoms, than the first and second trimesters. The likelihood of searching for help from family members would be more probable. Several possible explanations for this finding might be as follows: on the one hand, the affective tone/atmosphere in the family and closely accompanying family members in an environment of care would increase the willingness to express emotions and potentially affect psychological adjustment. On the other hand, mobilizing and obtaining more family resources could relieve emotional symptoms and enhance their self-efficacy, ability, and confidence to cope with problems. In fact, pregnant women mostly receive support from their mothers and husbands. For instance, maternal mothers and mothers-in-law might provide vicarious experiences relating to infant care and verbal encouragement, thus increasing maternal parental self-efficacy, and less stress was positively associated with marital support (53). In summary, the less a woman would experience stress and depression, the higher the probability that a mother would feel a bit more confident in resolving childcare problems related to new motherhood. The results suggested that it would be necessary to provide a supportive, stable, and harmonious family environment for a pregnant woman.

Nonetheless, the management of emotional problems during pregnancy remains a significant obstacle in China. First, under the traditional cultural background, pregnant women may not seek assistance because of stigma, which makes emotional problems not fully understood (54). A survey showed that due to restricted resources in Hunan province, access to high-quality and sufficient services for mental health management, treatment, and medical counseling was limited (55). Moreover, authoritative guidelines to manage mental health during pregnancy were poorly developed than Western developed countries (54). These phenomena indicated that improvement in family function could provide direct and rapid intervention for pregnant women to prevent the development of depression and anxiety symptoms.

The current study was a community-based study with low no-response rates; it might be generalized to other regions due to similar populations and socioeconomic backgrounds. Moreover, the current study was of great practical importance. Unlike Western developed countries, China's two-child family has been implemented since 2016. The policy aims to boost the country's total fertility rate (56). Thus, the population of pregnant women became high. Under complicated family and social culture, pregnant women would face various stress that leads to a series of depression and anxiety symptoms. For public health departments, merely considering family function or self-efficacy is not sufficient to explain their influence on each other. Expound researchers would not merely consider them; instead, they must consider a mutual association among family function, emotional symptoms, and self-efficacy.

There are several limitations to this study that should be considered. This cross-sectional study was limited by causal inferences. Anxiety and depression symptoms were measured based on the self-report of the last 2 weeks. To some extent, recall bias might have influenced the result. The findings showed that depression and anxiety symptoms were not completely mediated by the association between family function and self-efficacy. Other factors, such as social support (57), which contribute to the relationship between family function and self-efficacy, should further be explored in future studies. Lastly, in the current study, “emotional symptoms” was a shorthand way of saying “anxiety and depression symptoms.” Future work needs to focus on a composite anxiety and depression subscore that is then correlated with self-efficacy and family function.

## Conclusion

This study concluded that anxiety and depression symptoms mediated the relationship between family function and self-efficacy in pregnant women in the third trimester. The mediating effect of anxiety and depression symptoms between family functions and self-efficacy suggested that psychological intervention and support for pregnant women with serious family function might enhance their self-efficacy.

## Data Availability Statement

The original contributions presented in the study are included in the article/Supplementary Material, further inquiries can be directed to the corresponding authors.

## Ethics Statement

The studies involving human participants were reviewed and approved by the Ethics Committee of the Xiangya school of public Health (XYGW-2019-056), all of participants were volunteered for the survey and informed consent was signed by each participant during the investigation. The patients/participants provided their written informed consent to participate in this study.

## Author Contributions

ZW, ZB, YY, ZX, and HZ: data collection. ZW, ZX, and HZ: conceptualization. ZW and XX: data curation, writing, original draft preparation, methodology, software, and review. ZW, ZX, HZ, and LS: visualization and investigation. ZX, HZ, LS, XH, and XX: supervision. All authors contributed to the article and approved the submitted version.

## Funding

This study was financially supported by the National Natural Science Foundation of China (81701356), Major Scientific and Technological Projects for the Collaborative Prevention and Control of Birth Defects in Hunan Province (2019SK1015), the Science and Technology Programs of Changsha city (kq1801084), and Technology Projects of Administration of Traditional Chinese Medicine (201918).

## Conflict of Interest

The authors declare that the research was conducted in the absence of any commercial or financial relationships that could be construed as a potential conflict of interest.

## Publisher's Note

All claims expressed in this article are solely those of the authors and do not necessarily represent those of their affiliated organizations, or those of the publisher, the editors and the reviewers. Any product that may be evaluated in this article, or claim that may be made by its manufacturer, is not guaranteed or endorsed by the publisher.
