# Supplementary material for: Does the Presence of Anxiety and Depression Symptoms Mediate the Association Between Family Functions and Self-Efficacy in Pregnant Women in the Third Trimester?: A Community-Based Cross-Sectional Survey
Source: Front Psychiatry. 2021 Nov 4;12:726093. doi: 10.3389/fpsyt.2021.726093 (PMC8599816; doi:10.3389/fpsyt.2021.726093)
Supplement: Supplementary file 1 [file Data_Sheet_1.docx]

Appendix 1

**Stable 1 The mediation effects of anxiety symptoms between self-efficacy and family functions**

|  | **Dependent variable^*^** | | | | | | |
| --- | --- | --- | --- | --- | --- | --- | --- |
|  | **Path c** |  | **Path a** |  | **Path b** |  | **Inverse path** |
| **Variables** | GSES |  | Anxiety |  | GSES |  | APGAR |
| GSES |  |  |  |  |  |  | 0.936^***^(0.155)  95%CI: 0.632~1.240 |
| Anxiety |  |  |  |  | -0.016^***^(0.005)  95%CI: -0.026~ -0.006 |  | -0.131^***^(0.022)  95%CI: -0.174~ -0.088 |
| APGAR | 0.053^***^(0.008)  95%CI: 0.038~0.068 |  | -0.378^***^(0.054)  95%CI: -0.484~ -0.271 |  | 0.047^***^(0.008)  95%CI: 0.032~0.063 |  |  |
| Residence (ref=rural) | -0.024(0.045)  95%CI:-0.112 ~0.064 |  | 0.408(0.318)  95%CI: -0.216~1.032 |  | -0.018(0.044)  95%CI: -0.105~0.070 |  | -0.287(0.198)  95%CI: -0.675~0.101 |
| Marital status (ref=single/divorced/widowed) | 0.053(0.092)  95%CI: -0.127~0.233 |  | -1.027(0.651)  95%CI: -2.305~0.251 |  | 0.037(0.091)  95%CI:-0.142 ~0.215 |  | 0.317(0.405)  95%CI: -0.479~1.113 |
| Age between 30-34 (ref=≥35) | 0.003(0.063)  95%CI: -0.122~0.128 |  | 0.690(0.452)  95%CI: -0.197~1.576 |  | 0.041(0.063)  95%CI: -0.110~0.138 |  | -0.343(0.281)  95%CI: -0.895~0.208 |
| Age between 25-29 (ref=≥35) | -0.074(0.062)  95%CI:-0.195 ~0.046 |  | 0.806(0.438)  95%CI: -0.054~1.665 |  | -0.062(0.061)  95%CI: -0.182~0.059 |  | -0.167(0.273)  95%CI: -0.703~0.368 |
| Age ≤24 (ref=≥35) | -0.035(0.079)  95%CI: -0.189~0.120 |  | 1.153**(0.561)  95%CI:0.052 ~2.254 |  | -0.016(0.079)  95%CI:-0.171 ~0.138 |  | -0.421(0.349)  95%CI:-1.107 ~0.265 |
| Employment (ref=not) | 0.100**(0.046)  95%CI: 0.009~0.192 |  | -0.513(0.330)  95%CI:-1.161 ~0.135 |  | 0.092**(0.046)  95%CI:0.002 0.183~ |  | -0.238(0.206)  95%CI: -0.643~0.166 |
| Education level was high school (ref= junior school or below) | -0.023(0.058)  95%CI: -0.137~0.091 |  | -0.428(0.414)  95%CI: -1.240~0.384 |  | -0.030(0.058)  95%CI: -0.144~0.084 |  | 0.020(0.258)  95%CI: -0.485~0.526 |
| Education level was college or above(ref=junior school or below ) | 0.052(0.057)  95%CI: -0.060~0.163 |  | 0.046(0.404)  95%CI:-0.746 ~0.838 |  | 0.052(0.056)  95%CI:-0.058 ~0.163 |  | 1.008***(0.249)  95%CI: 0.520~1.496 |
| Per-capita monthly income between 3001-7999 (ref=≤3000 RMB) | -0.005(0.067)  95%CI: -0.137~0.126 |  | -0.822(0.477)  95%CI: -1.758~0.114 |  | -0.018(0.067)  95%CI:-0.149 ~0.113 |  | -0.624**(0.296)  95%CI: -1.206~-0.042 |
| Per-capita monthly income >8000 (ref= ≤3000 RMB) | 0.172**(0.077)  95%CI:0.021 ~0.322 |  | -0.774(0.545)  95%CI: -1.844~0.296 |  | 0.159**(0.076)  95%CI:0.010 ~0.309 |  | -0.071(0.340)  95%CI:-0.739 ~0.597 |
| Smoking status was quit (ref=yes) | 0.252(0.316)  95%CI: -0.368~0.872 |  | -3.348(2.247)  95%CI: -7.759~1.064 |  | 0.199(0.315)  95%CI: -0.419~0.816 |  | 1.477(1.399)  95%CI: -1.268~4.222 |
| Smoking status was never (ref=yes) | 0.411(0.306)  95%CI:-0.191 ~1.012 |  | -4.355**(2.179)  95%CI: -8.633~-0.077 |  | 0.341(0.305)  95%CI:-0.258 ~0.941 |  | 1.996(1.358)  95%CI:-0.669 ~4.661 |
| Alcohol consumption at the present was no (ref=yes) | -0.044(0.063)  95%CI:-0.167 ~0.079 |  | -0.397(0.446)  95%CI: -1.273~0.479 |  | -0.050(0.062)  95%CI: -0.173~0.072 |  | 0.410(0.277)  95%CI:-0.135 ~0.954 |
| Exercising at the present (ref=no) | 0.036(0.067)  95%CI: -0.095~0.168 |  | -0.594(0.477)  95%CI: -1.531~0.342 |  | 0.027(0.067)  95%CI:-0.104 ~0.158 |  | 0.298(0.297)  95%CI: -0.485~0.526 |
| History of abortion (ref=no) | -0.039(0.050)  95%CI:-0.137 ~0.059 |  | 0.707**(0.355)  95%CI: 0.011~1.404 |  | -0.028(0.050)  95%CI:-0.125 ~0.070 |  | 0.329(0.221)  95%CI: -0.105~0.763 |
| 1 of child in the family (ref=0) | 0.042(0.050)  95%CI: -0.057~0.140 |  | -0.235(0.358)  95%CI: -0.937~0.467 |  | 0.038(0.050)  95%CI: -0.060~0.136 |  | -0.051(0.222)  95%CI: -0.487~0.386 |
| 2 of child in the family (ref=0) | 0.120(0.086)  95%CI: -0.048~0.289 |  | 0.202(0.611)  95%CI: -0.998~1.402 |  | 0.124(0.085)  95%CI:-0.044 ~0.291 |  | -0.256(0.381)  95%CI:-1.003 ~0.491 |
| Pregnancy complications(ref=yes) | -0.008(0.062)  95%CI:-0.129 ~0.113 |  | -0.582(0.440)  95%CI:-1.445~0.281 |  | -0.017(0.062)  95%CI:-0.138 ~0.104 |  | -0.067(0.274)  95%CI:-0.604 ~0.470 |
| Regular obstetrical examination during pregnancy (ref=no) | 0.033(0.069)  95%CI: -0.102~0.167 |  | 0.255(0.489)  95%CI: -0.704~1.214 |  | 0.037(0.068)  95%CI:-0.098 ~0.171 |  | 1.159***(0.301)  95%CI: 0.568~1.751 |
| Constant | 2.390^***^(0.165) |  | 7.792^***^(1.170) |  | 2.514^***^(0.168) |  | 4.914^***^(0.829) |
| Observations | 813 |  | 813 |  | 813 |  | 813 |
| R^2^ | 0.139 |  | 0.111 |  | 0.150 |  | 0.218 |
| Adjusted R^2^ | 0.117 |  | 0.089 |  | 0.127 |  | 0.197 |
| Residual Std. Error | 0.520  (df = 792) |  | 3.699  (df = 792) |  | 0.517  (df = 791) |  | 2.300  (df = 791) |
| F Statistic | 6.375^***^  (df = 20; 792) |  | 4.953^***^  (df = 20; 792) |  | 6.634^***^  (df = 21; 791) |  | 10.502^***^  (df = 21; 791) |

^*^*Note:**p<0.1; **p<0.05; ***p<0.01

Appendix 2

**Stable 2 The mediation effects of depression symptoms between self-efficacy and family functions**

|  | **Dependent variable^*^** | | | | | | |
| --- | --- | --- | --- | --- | --- | --- | --- |
|  | **Path c** |  | **Path a** |  | **Path b** |  | **Inverse path** |
| **Variables** | GSES |  | Depression |  | GSES |  | APGAR |
| GSES |  |  |  |  |  |  | 0.890^***^(0.156)  95%CI: 0.584~1.195 |
| Depression |  |  |  |  | -0.024^***^(0.006)  95%CI: -0.035~ -0.013 |  | -0.152^***^(0.024)  95%CI: -0.200~ -0.104 |
| APGAR | 0.053^***^(0.008)  95%CI: 0.038~0.068 |  | -0.360^***^(0.049)  95%CI: -0.455~ -0.265 |  | 0.045^***^(0.008)  95%CI: 0.029~0.060 |  |  |
| Residence (ref=rural) | -0.024(0.045)  95%CI:-0.112 ~0.064 |  | 0.378(0.284)  95%CI: -0.180~0.936 |  | -0.015(0.044)  95%CI: -0.102~0.072 |  | -0.283(0.197)  95%CI: -0.670~0.105 |
| Marital status (ref=single/divorced/widowed) | 0.053(0.092)  95%CI: -0.127~0.233 |  | -0.535(0.582)  95%CI:-1.677 ~0.608 |  | 0.040(0.091)  95%CI:-0.138 ~0.218 |  | 0.371(0.404)  95%CI:-0.422 ~1.165 |
| Age between 30-34 (ref=≥35) | 0.003(0.063)  95%CI: -0.122~0.128 |  | 0.137(0.404)  95%CI: -0.655~0.930 |  | 0.006(0.063)  95%CI: -0.117~0.130 |  | -0.411(0.280)  95%CI: -0.961~0.138 |
| Age between 25-29 (ref=≥35) | -0.074(0.062)  95%CI:-0.195 ~0.046 |  | 0.329(0.391)  95%CI: -0.439~2.854 |  | -0.066(0.061)  95%CI: -0.186~0.053 |  | -0.225(0.272)  95%CI: -0.759~0.309 |
| Age ≤24 (ref=≥35) | -0.035(0.079)  95%CI: -0.189~0.120 |  | 1.045**(0.502)  95%CI: 0.060~2.030 |  | -0.009(0.078)  95%CI:-0.163 ~0.144 |  | -0.413(0.349)  95%CI: -1.098~0.271 |
| Employment (ref=not) | 0.100**(0.046)  95%CI: 0.009~0.192 |  | -0.202(0.295)  95%CI: -0.782~0.377 |  | 0.096**(0.046)  95%CI: 0.005~0.186 |  | -0.197(0.205)  95%CI: -0.600~0.206 |
| Education level was high school (ref= junior school or below) | -0.023(0.058)  95%CI: -0.137~0.091 |  | -0.007(0.370)  95%CI: -0.733~0.719 |  | -0.023(0.058)  95%CI:-0.136 ~0.089 |  | 0.074(0.257)  95%CI: -0.430~0.578 |
| Education level was college or above(ref=junior school or below ) | 0.052(0.057)  95%CI: -0.060~0.163 |  | 0.402(0.361)  95%CI: -0.307~1.110 |  | 0.061(0.056)  95%CI:-0.049 ~0.172 |  | 1.062***(0.248)  95%CI: 0.575~1.549 |
| Per-capita monthly income between 3001-7999 (ref=≤3000 RMB) | -0.005(0.067)  95%CI: -0.137~0.126 |  | -0.802(0.426)  95%CI: -1.639~0.035 |  | -0.025(0.066)  95%CI: -0.155~0.106 |  | -0.617***(0.296)  95%CI: -1.218~-0.056 |
| Per-capita monthly income >8000 (ref= ≤3000 RMB) | 0.172**(0.077)  95%CI:0.021 ~0.322 |  | -0.383(0.487)  95%CI:-1.340 ~0.574 |  | 0.162**(0.076)  95%CI: 0.014~0.311 |  | -0.020(0.340)  95%CI:-0.687 ~0.646 |
| Smoking status was quit (ref=yes) | 0.252(0.316)  95%CI: -0.368~0.872 |  | -0.428(2.009)  95%CI: -4.372~3.516 |  | 0.242(0.312)  95%CI: -0.371~0.855 |  | 1.855(1.394)  95%CI: -0.882~4.592 |
| Smoking status was never (ref=yes) | 0.411(0.306)  95%CI:-0.191 ~1.012 |  | -0.971(1.949)  95%CI: -4.794~2.854 |  | 0.387(0.303)  95%CI: -0.208~0.982 |  | 2.428(1.352)  95%CI:-0.225 ~5.082 |
| Alcohol consumption at the present was no (ref=yes) | -0.044(0.063)  95%CI:-0.167 ~0.079 |  | -0.110(0.399)  95%CI:-0.894 ~0.673 |  | -0.046(0.062)  95%CI: -0.168~0.075 |  | 0.441(0.277)  95%CI:-0.102 ~0.985 |
| Exercising at the present (ref=no) | 0.036(0.067)  95%CI: -0.095~0.168 |  | -1.044**(0.427)  95%CI: -1.881~-0.206 |  | 0.011(0.067)  95%CI:-0.120 ~0.142 |  | 0.218(0.297)  95%CI:-0.366 ~0.801 |
| History of abortion (ref=no) | -0.039(0.050)  95%CI:-0.137 ~0.059 |  | 0.155(0.317)  95%CI: -0.468~0.778 |  | -0.035(0.049)  95%CI: -0.132~0.062 |  | 0.258(0.220)  95%CI: -0.175~0.690 |
| 1 of child in the family (ref=0) | 0.042(0.050)  95%CI: -0.057~0.140 |  | 0.308(0.320)  95%CI: -0.320~0.935 |  | 0.049(0.050)  95%CI: -0.048~0.147 |  | 0.029(0.222)  95%CI: -0.407~0.465 |
| 2 of child in the family (ref=0) | 0.120(0.086)  95%CI: -0.048~0.289 |  | 0.263(0.547)  95%CI:-0.810 ~1.336 |  | 0.127(0.085)  95%CI: -0.040~0.294 |  | -0.237(0.380)  95%CI:-0.983 ~0.509 |
| Pregnancy complications(ref=yes) | -0.008(0.062)  95%CI:-0.129 ~0.113 |  | -0.439(0.393)  95%CI:-1.211 ~0.332 |  | -0.019(0.061)  95%CI: -0.139~0.101 |  | -0.058(0.273)  95%CI: -0.594~0.479 |
| Regular obstetrical examination during pregnancy (ref=no) | 0.033(0.069)  95%CI: -0.102~0.167 |  | -0.435(0.437)  95%CI: 1.293~0.423 |  | 0.022(0.068)  95%CI: -0.111~0.155 |  | 1.058***(0.301)  95%CI: 0.466~1.649 |
| Constant | 2.390^***^(0.165) |  | 10.158^***^(1.046) |  | 2.636^***^(0.172) |  | 5.528^***^(0.853) |
| Observations | 813 |  | 813 |  | 813 |  | 813 |
| R^2^ | 0.139 |  | 0.108 |  | 0.159 |  | 0.220 |
| Adjusted R^2^ | 0.117 |  | 0.085 |  | 0.137 |  | 0.200 |
| Residual Std. Error | 0.520  (df = 792) |  | 3.307  (df = 792) |  | 0.514  (df = 791) |  | 2.296  (df = 791) |
| F Statistic | 6.375^***^  (df = 20; 792) |  | 4.789^***^  (df = 20; 792) |  | 7.127^***^  (df = 21; 797) |  | 10.650^***^  (df = 21; 791) |

*Note:**p<0.1; **p<0.05; ***p<0.01
